# Tasting the Italian Terroir through Craft Beer: Quality and Sensory Assessment of Cascade Hops Grown in Central Italy and Derived Monovarietal Beers

**DOI:** 10.3390/foods10092085

**Published:** 2021-09-03

**Authors:** Katya Carbone, Giulia Bianchi, Maurizio Petrozziello, Federica Bonello, Valentina Macchioni, Barbara Parisse, Flora De Natale, Roberta Alilla, Maria Carla Cravero

**Affiliations:** 1 CREA Research Centre for Olive, Fruit and Citrus Crops, Via di Fioranello 52, 00134 Rome, Italy; valentina.macchioni14@gmail.com; 2 CREA Research Centre for Engineering and Agro-Food Processing, Via G. Venezian 26, 20133 Milan, Italy; giulia.bianchi@crea.gov.it; 3 CREA Research Centre for Viticulture and Enology, Via P. Micca 35, 14100 Asti, Italy; maurizio.petrozziello@crea.gov.it (M.P.); federica.bonello@crea.gov.it (F.B.); mariacarla.cravero@crea.gov.it (M.C.C.); 4 CREA Research Centre for Agriculture and Environment, Via della Navicella 4, 00184 Rome, Italy; barbara.parisse@crea.gov.it (B.P.); flora.denatale@crea.gov.it (F.D.N.); roberta.alilla@crea.gov.it (R.A.)

**Keywords:** *terroir*, Cascade hop, *Humulus lupulus* L., single hop beers, sensory analysis

## Abstract

The present study aimed to chemically and sensorially characterize hop samples, cv Cascade, grown in two different Italian regions (Latium and Tuscany) as well as their derived beers by a multi analytical approach. Significant differences in bitter acid, oil and polyphenol content were observed for hop samples according to their origin. Gas chromatography-olfactometry analysis pointed out floral notes for Tuscany samples, where hops from Latium were characterized by spicy and resinous notes, correlated to the presence of sesquiterpenes. Differences in the molecular fingerprinting were also highlighted by Fourier–Transform Infrared Spectroscopy. The differences found in the hops were reflected in the beers, which were clearly recognized as distinct by a sensory panel. Both beer samples were mainly characterized by six aroma compounds (linalool, geraniol and β-damascenone, citronellol, 2-phenylethyl acetate, and 2-phenylethanol), three of which were potentially responsible for the geographic origin of the hops given their significantly different concentrations.

## 1. Introduction

Many crops display differential geographic phenotypes and sensorial signatures, encapsulated by the concept of *terroir*. This has been primarily used to link wine production to specific places. The term *terroir* increasingly refers to ecological and cultural conditions that create a sense of group identity by engaging in and consuming particular products, mainly food and beverages, leading in recent years to a growing demand for local artisanal food products worldwide [1]. The enhancement of the concept of *terroir* in some sectors of the agrifood industry, first of all that of wine, has led over the years to the creation of protection and/or regional brands that have boosted rural development and the rural economy, have helped to create a recognizable system of a territory’s unique characteristics through the development of its product specialization and the expansion of potential markets for regional products and services [2]. All of this can easily be reflected in territory branding, which can be used as a stimulus for rural survival and to increase the potential of sustainable development of the internal rural areas. According to the American Marketing Association (AMA), a “brand” is “a word, sign, symbol or design solution, or a combination thereof, created for the purpose of designating the goods and services of a particular seller or group of sellers to distinguish them from competitors” [2]. In this sense, emerging agrifood sectors, such as the craft beer one, can benefit greatly by linking their production to the concept of *terroir*, establishing trusting relationships with the consumer, communicating diversity, quality, authenticity, links with the landscape and cultural identities of the product to increase competitiveness and uniqueness with respect to industrial brands [2]. Craft beer, in fact, much more than other products, reflects local styles or ingredients. The movement of Italian craft beer was born in 1996, drawing from the concepts of territoriality, nature and authenticity in its marketing, but, currently, most of the raw materials used in the production of these beers are imported [3]. However, in the last few years, trying to follow the path already well traced by the national wine sector from which the concepts of terroir and branding of the agri-product as an added value were generated, small agricultural realities have started to flourish, capable of producing malted barley and especially hops, destined to the production of 100% local beers [4]. Several studies have been published about the influence of *terroir* on hops [5,6,7]. However, to the best of our knowledge, only two studies are actually present in literature about the application of the *terroir* concept to the brewing value of hops as reflected in the chemical and sensory features of the related craft beer produced from it [5,8]. Literature studies have highlighted a significant influence of the growing region on the quality traits of Cascade hops cultivated in Italy [6,9]. However, none of them reported any data on brewing applications with these hops. In light of these considerations, the main purpose of the present study was to evaluate the influence of *terroir* in craft beers produced with Cascade hops from two different Italian regions. The hop samples were analyzed for their molecular fingerprinting by Fourier–Transform Infrared Spectroscopy (FTIR), bitter acids, cohumulone and oil content as well as for their olfactometric profile by gas chromatography-olfactometry (GC-O) of the hop hydrodistillates, previously characterized by GC-MS. Then, to evaluate the quality characteristics imparted by the addition of hops, the characterizations of olfactometric, analytical and sensory profiles of the experimental beers were carried out.

## 2. Materials and Methods

### 2.1. Chemicals

All used reagents were of analytical spectrophotometric grade (VWR; Milan, Italy). Bitter acid mixture standard (International calibration extract, ICE-3) was purchased from Labor Veritas Co. (Zurich, Switzerland). ICE-3 was reported to contain 13.88% cohumulone and 30.76% of n-humulone + adhumulone (α-acids), and 13.44% of colupulone and 10.84% of n-lupulone + adlupulone (β-acids). All GC-MS standards were purchased, except where specified, from Merck Corporation (Darmstadt, Germany) at the maximum available purity grade. The water employed was previously purified in a Milli-Q system (Millipore, Milan, Italy). 0.45-μm pore size membrane filters from Pall (Pall Italia, Milan, Italy) were used for filtration of both mobile phases and samples.

### 2.2. Plant Material

Cascade hop samples used for brewing trials were obtained from two organic hop farms, located in the Latium (CAS_L) and Tuscany (CAS_T) regions, whose details are given in Table 1. All hop cones used in the present study were collected at commercial maturity from 4 years-old plants (vintage 2018). Hop plants were grown on six-meter-high trellises. The planting system, for both farms, featured rows 3 m apart with a 0.8 m distance within each row. Standard organic farming practices were carried out on both farms. After harvest, cones were dried to 12–13% moisture in hop kilns located at each site, at 52–55 °C for about 8–12 h. Once dried, cones were cooled down for about 12–24 h, then vacuum-packed in aluminum triple bag and kept at 4 °C until use.

### 2.3. Meteorological Data

The global meteorological dataset from surface reanalysis ERA5-Land (E5L; https://cds.climate.copernicus.eu/cdsapp#!/dataset/reanalysis-era5-land?tab=overview, accessed on 4 August 2021) was selected for the current study. E5L has a spatial resolution of approximately 9 km and is freely available as product of the Copernicus Climate Change Service (C3S). E5L weather data were provided with an hourly time-step and released with a delay of 2–3 months before the present. The analysis was based on two variables derived from this dataset: air temperature at 2 m asl (°C; T2) and total precipitation (mm; TP). The E5L grid cells, which correspond to the farms’ locations, were selected. Hourly data for the period from October 2016 to September 2017 were temporally aggregated at monthly scale, based on the mean and the sum for temperature and precipitation, respectively. Data processing was performed through the R software (https://www.R-project.org, accessed on 4 August 2021).

### 2.4. Hop Quality Traits

#### 2.4.1. Molecular Fingerprinting Analysis and Evaluation of Terroir Effect

In the present study, infrared spectroscopy was used to evaluate hop quality traits and to investigate the role of *terroir* on the hop molecular fingerprinting, by coupling spectral information acquired in the mid-infrared (MIR) with chemometric tools. Attenuated Total Reflectance (ATR) FTIR spectra of hop dried cones were collected using a iS 50 Nicolet FTIR spectrometer (Thermo Fisher Scientific Inc., Milan, Italy), according to Macchioni et al. [10] without modifications. Spectra were acquired at room temperature and then processed with the OMNIC™ software (Thermo Fisher Scientific Inc., Milan, Italy). 

#### 2.4.2. Chemical Analysis and Total Bitter Acid Content

Hop samples used for brewing trials were analyzed for moisture content according to EBC method 7.2 [11]. Data were reported as percentage on dry basis (db). Hop storage index (HIS) was measured according to ASBC method Hops-12 [12]. The bitter acid content of hop samples was determined on 2.5 g of ground hop cones, using toluene as extraction solvent, according to the official ASBC Hops-6 method [12]. α- and β-acid content was determined spectrophometrically at 275, 325 and 355 nm; results were the average of three independent measurements and data were expressed as % *m*/*m* on dry basis (db). 

#### 2.4.3. Determination of Individual Bitter Acids by HPLC

Hop bitter acids were separated and identified by an analytical HPLC system (Agilent 1100 series, Agilent, Milan, Italy), equipped with a diode array detector (DAD; Agilent Technologies, Milan, Italy), according to the international ASBC Hops-14 method as reported by Carbone et al. [13] without modifications.

The injection volume was 50 μL and the samples were membrane filtered (Millipore PTFE 0.45 mm, Milan, Italy) prior to the HPLC analysis. The separation was performed on a Synergi C18 column (Phenomenex, 4.6 × 150 mm; 4 μm particle size, set at 40 °C). Chromatograms were acquired at 326 nm. For the quantification of α- and β-acids, a calibration curve was obtained from dilution of ICE-3 standard. The results were expressed as % *m*/*m* on dry basis (db).

#### 2.4.4. Total Oil Content

Hop essential oils (HEO) were extracted by hydro-distillation with a Clevenger apparatus, according to the ASBC Hops-13 method [12], for 4 h. Results are the average of three independent extractions and data were expressed as mL 100 g^−1^ on dry basis (db).

#### 2.4.5. Gas Chromatography-Olfactometry (GC-O) Analysis of HEO

**Figure 1:**
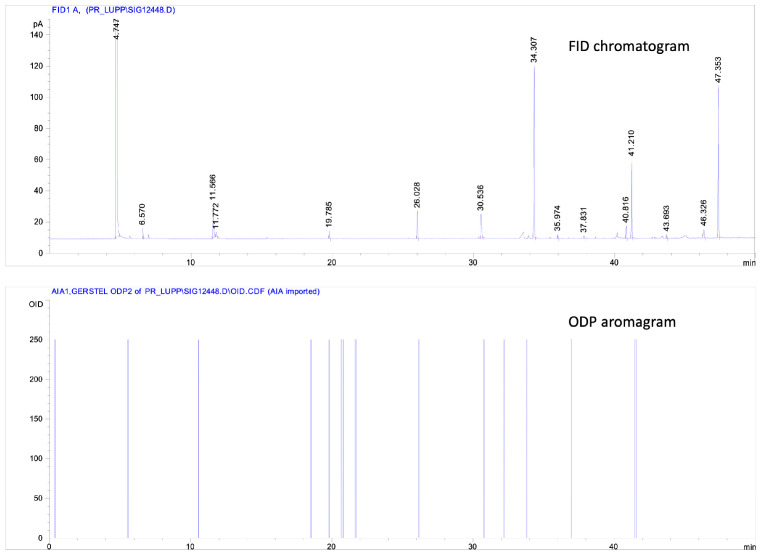
A representative aromagram from Gas chromatography-olfactometry (GC-O) analysis of hop samples analyzed.

GC–O is a hyphenated technique that allows the simultaneous analysis of volatile fractions by an instrumental detector and the human nose. This allows us to separate and recognize the “odor active” fractions, which contribute to the odor of the extract. The result of this analysis is a double plot called “aromagram” (Figure 1), which represents the response of both detectors, chromatographic peaks and sensory responses, indicated as OE (odor event). GC-O analyses were performed at CREA-IT laboratory, located in Milan. The system used was composed of an Agilent 6890 N GC equipped with an FID and a DB-1 capillary column (60 m × 0.25 mm i.d., 0.25 μm film thickness). Helium was used as carrier gas (1.3 mL min^−1^). The injector and FID temperatures were set at 250 °C and the following column temperature program was applied: 40 °C for 5 min, 2.5 °C min^−1^ up to 160 °C held for 5 min (duration: 58 min). The eluate was split 1:1 at the column outlet, linked to an olfactometric system that included the Olfactory Detector Port ODP2 Gerstel (Gerstel GmbH) equipped with the ODPneumatics module to control humidification and make up gas flows. The analyses were performed by using a direct intensity method described in [14]. The olfactometric data (intensity, duration and area of each odor event, OE) were collected through a potentiometer with the ODP recorder integrated with the GC software Chemstation Rev A 10.02. The area of each OE was calculated by the software from the intensity and duration values and shown as a chromatographic peak. The GC-O results were expressed as OE areas (A, average) and maximum intensity (Imax, median). The panel was composed of 7 panelists (1 male and 6 female), aged between 35 and 50 years, who were familiarized with the products. Before the analysis of the samples, all panelists attended two training sessions to learn to identify the main odor categories present in hop and beer products, using one representative compound for each of them. Solutions at different concentrations of the following standards were used: ethyl hexanoate (fruit), citronellol (citrus, flower), β-myrcene (resinous), α-humulene (woody), mesifurane (caramel). For each GC–O analysis, 2 panelists were involved, sniffing was divided into two parts of 25 min and each panelist participated in the sniffing of both parts. For hop analysis, oil was diluted in hexane (1:2 *v*/*v*) and 1.5 µL were injected in split mode (split flow: 12 mL min^−1^, split ratio 1:10). GC-O results were related to composition data resulting from the GC-MS of HEO (Table 2). 

GC-MS was performed using an Agilent 5973N MSD connected to an Agilent 6890 GC, with the same column and chromatographic conditions of GC-O. The MS settings were as follows: filament voltage, 70 eV; scan range, *m*/*z* 45–800; scan speed, 1.4 scan/s; injector and interface temperature, 250 °C. Identification was performed by comparing mass spectra with those stored in databases (NIST 08 and Wiley 7 libraries), and comparing their Kováts indices, calculated using n-alkanes reference hydrocarbons, with tabulated Kováts indices.

### 2.5. Brewing Trials

Two experimental beers with Italian Cascade hop dried cones were produced at ‘Opificio birrario’, an Italian craft brewery, on a semi-industrial scale plant (2.5 hL) using the single-hop technology. This implied that identical recipes and raw materials were used, and the only variable in the brewing process was the choice of the geographical origin of Cascade hop. Batches of different experimental beers were produced by following the brewery’s own recipe used to produce one of its commercial beers and therefore it cannot be reported herein in detail. Briefly, 100% Pilsner malt and a ‘neutral’ industrial top fermenting yeast were selected to keep malt and yeast aromas in the background, while the hoppy character was brought to the foreground. Hop additions were identical for the two beers and standardized by weight: 200 g of hops per hL first-wort hopping (at the start of boiling), 200 g of hops per hL middle hopping and 200 g of hops per hL late hopping (at the end of boiling; 20 IBU) and 450 g of hops per hL in dry hopping (added after three days from the start of fermentation). The beers were stored at 1 °C until analysis.

### 2.6. Beer Quality Traits

#### 2.6.1. Gas Chromatography-Mass Spectrometry (GC-MS) Analysis

We added together 200 mL of each beer, which was diluted 2-fold, and 2 mL of 1-heptanol (77.18 mg L^−1^) as internal standard; the mixture was loaded onto a reversed-phase C18 EC cartridge (5 g; Biotage AB, Uppsala, Sweden), previously activated with 20 mL of methanol and 75 mL of water. After washing with water (30 mL), the free volatile substances were eluted with 30 mL of dichloromethane HPLC grade; the organic phase was dried with the addition of anhydrous sodium sulphate, concentrated by evaporation and analyzed by GC-MS. GC-MS analysis was carried out by an Agilent 7890 Series gas chromatograph (Agilent Technologies, Inc., Santa Clara, CA, USA), equipped with an Agilent 5975N Mass Selective Detector (MSD). The concentrate (1 μL) was analyzed on a Zebron ZB-WAX column (60 m × 0.25 mm i.d., 0.25 μm film thickness; Phenomenex, Torrance, CA, USA). Conditions were as follows: helium was used as a carrier gas, with a constant flow of 1 mL min^−1^. The source and the transfer line were kept at 230 °C, and the injector at 250 °C. The oven temperature was kept at 45 °C for 2 min, then increased to 60 °C at a rate of 30 °C min^−1^, further increased from 60 °C to 160 °C at a rate of 2 °C min^−1^, lastly, from 160 °C to 230 °C at a rate of 3 °C min^−1^ and kept at 230 °C for 15 min. The acquisition of mass spectra was carried out in total ion current mode from 29 to 300 m/z, and the area of each peak was measured using the ChemStation software (Agilent Technologies, Santa Clara, CA, USA). Identification was carried out comparing mass spectra and retention times with those of the authentic standards, where available. The concentration was calculated as μg 1-heptanol (internal standard) equivalents per L.

#### 2.6.2. GC-O Analysis

The analysis of beer samples was carried out in the same experimental conditions described for HEO ones. Samples were prepared using the headspace solid phase microextraction technique (HS-SPME). Each replicate was made up by 8 mL of beer with 2 g of NaCl added, put in a 20 mL glass vial closed with an aluminum cap with silicone-rubber septum. The extraction of volatile compounds was performed using a DVB/CAR/PDMS fiber (absorption step: 45 °C for 30 min; desorption step in the injector port: 250 °C for 5 min in splitless). HS-SPME GC-MS trials on the same chromatographic conditions, as described in Section 2.4.5, were performed to recognize the main odor active fractions (Table 3).

#### 2.6.3. Sensory Analysis

Beers were evaluated by a trained panel (16 assessors: 8 females and 8 males, age 25–60) of the CREA Research Centre for Viticulture and Enology (Asti). The sensory tests were carried out in a tasting room (ISO norms 8589-2007). Beer samples (50 mL) were identified with a 3-digit code and poured in tasting glasses (ISO 3591-1977).

The tetrad test was utilized to discriminate the two samples, according to their similarity [15].

The beer sensory profiles were realized by the same panel but with 14 assessors, as two subjects: one male (age 43) and one female (age 58) were not available, following a procedure derived from the ISO standards (11035-1994) and applied in wine [16,17]. 

The first evaluation was a qualitative description of the products. The panel chose the attributes on their experience and with the help of a predefined list, realized with consideration of the literature [18]; http://beeraromawheel.com (accessed on 21 August 2021). 

A frequency threshold for the attribute citations was established: the attributes of color, taste, and mouthfeel were chosen when their frequency of identification by the panel was greater than “(number of assessors × number of wines)/2”. Regarding odor, its description is generally more complex: the 3rd-level descriptors were chosen when their frequency of identification was higher than “(number of assessors × number of wines)/4”. This procedure is similar to those applied by other authors in wine [19,20]. 

All the selected attributes were confirmed and discussed by the panel with suitable standards: orange blossom (orange blossom aroma for sweets), rose (rose extract), grapefruit (grapefruit fruit juice), pear (pear fruit juice), apple (apple fruit juice), canned green beans (canned green bean preserving liquid), hay-straw (hay and wheat straw), honey (wildflower honey), yeasts (dry *Saccharomyces cerevisiae* yeasts) and caramel/toasted (caramelized sugar mixed with toasted oak wood chips). The standard for the descriptor spicy was a mixture of spices (cloves, pepper, cinnamon, and nutmeg). A tasting sheet was created to measure the intensity of each chosen descriptor, using an unstructured intensity scale presented on a wheel. Three replicates of each beer sample were sensory analyzed by the panel.

### 2.7. Statistical Analysis

Statistical analysis on hop composition was performed with SPSS 25.0 software (SPSS, Inc., Chicago, IL, USA). Data were reported as mean ± standard deviation (SD) of three independent experiments with three replicates. Prior to chemometric applications, all variables, used for hop quality analysis, were auto scaled (transformation into z-scores) to standardize the statistical importance of all responses. Significant mean differences were established using the Mann–Whitney test for independent and nonparametric procedures (*p* < 0.0167 for Bonferroni’s correction, where not specified differently). 

The chemometric analysis of IR data, acquired from hop samples from farms under investigation during three consecutive years (2016–2018 harvest), was preceded by several pre-processing steps for each data set. The data obtained were then subjected to principal component analysis (PCA). This multivariate technique is usually the first step in data exploration: PCA defines new variables, which consist of linear combinations of the original variables, so that the first axis is in the direction that contains most of the variations. The Savitzky–Golay method with third-order smoothing polynomial through eleven points was used to calculate the second derivative of the IR spectra of hop samples used in the brewing trials (2018 vintage) to obtain a more detailed information on their molecular fingerprinting.

Statgraphics software ver. 5.1 (Manugistics, Rockville, MD, USA) was used to perform the ANOVA (LSD test, *p* ≤ 0.05) on GC–O OE areas, while the quantitative measurements from sensory analysis were subjected to ANOVA and the Tukey test (95%), using XLSTAT software version 2016 (Addinsoft, Paris, France).

## 3. Results

### 3.1. Preliminary Evaluation of the Impact of Growing Area on Hop Samples by Means of Vibrational Spectroscopy

**Figure 2:**
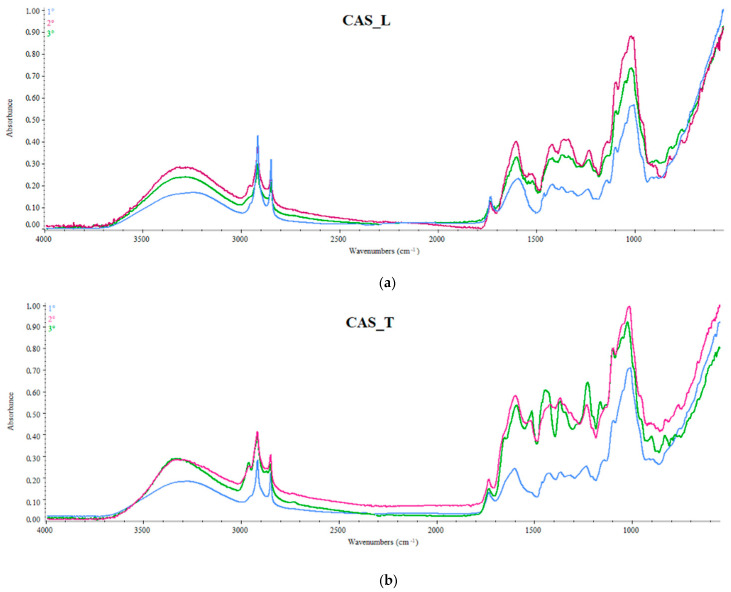
FT-IR raw spectra of hop samples from different growing area, collecting over three consecutive years: blue line: harvest 2016; red line: harvest 2017; green line: harvest 2018. (**a**) CAS_L: Cascade hop samples from Latium; (**b**) CAS_T: Cascade hop samples from Tuscany.

**Figure 3:**
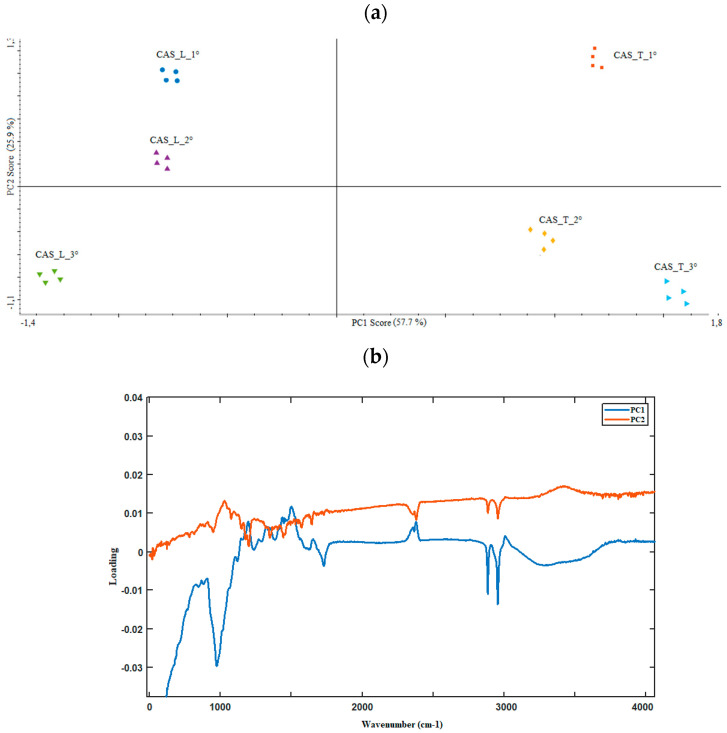
(**a**) PCA score plot of hop samples from different locations based on FTIR data matrix; (**b**) Loading plot of the spectral variables most contributing to the separation of hop samples on PC1 and PC2.

FTIR spectroscopy is a technique widely used in the determination of the authenticity and geographical traceability of agrifood products [21] but, as far as we know, it has never been applied in authenticating the geographical origin of hops. Besides, Paliotta et al. [22], for the first time, evaluated the potential of near infrared spectroscopy in the classification of hop samples according to genotype and origin. Here, to screen for differences or similarities quickly and easily in the overall fingerprinting of Cascade hop samples from the two different geographic regions considered in the brewing trials, the FTIR spectra of hops collected over three consecutive years were evaluated by PCA. The results highlighted spectral differences between hop samples analyzed, mainly in the region of the molecular signature (below 1800 cm^−1^; Figure 2). On the basis of eigenvalues > 1 (Kaiser’s criterion) and of the scree plot (not shown), two principal components (PCs), accounting for about 84% of the data matrix variance, were considered significant and allowed us to group the samples according to their different growing area along PC1 (Figure 3a). Based on the loading plot (Figure 3b), the variables contributing the most to the separation on PC1 and PC2 were absorbance values at λ: 2964, 2888, 1724 and 986 cm^−1^. The results point out that FTIR screening, combined with chemometric analysis, can differentiate the samples analyzed in relation to their geographical origin, suggesting a *terroir* effect on the molecular fingerprint of the hop samples coming from the two investigated farms. These findings agree with literature studies [6,8,9]. Based on this experimental evidence, we wanted to investigate if these differences were also transferable to beers produced with these hops. To this end, we conducted the brewing tests on the 2018 hop harvest, reporting below the results obtained on both hop cones and derived beers.

### 3.2. Brewing Trials: Evaluation of Growing Area on Hop Quality Traits 

**Figure 4:**
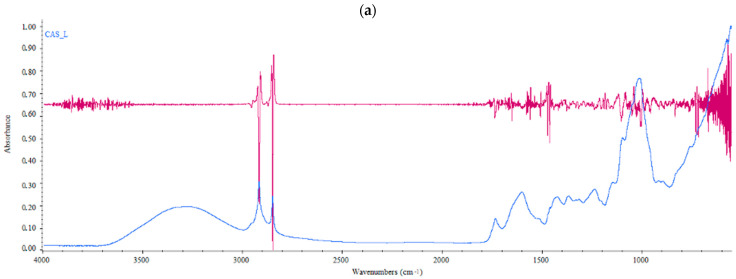

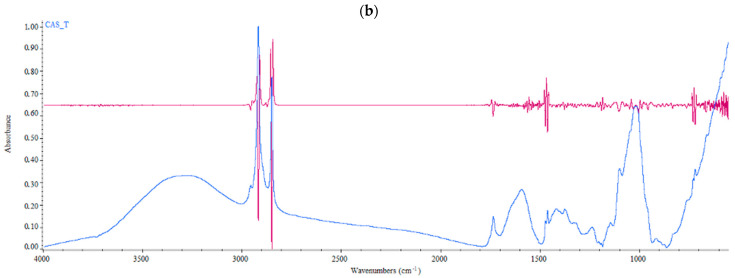
FT-IR raw (blue line) and derivative spectra (red line) of hop samples analyzed: (**a**) Latin hop sample (CAS_L) spectra; (**b**) Tuscan hop sample (CAS_T) spectra.

Figure 4 shows the FTIR molecular fingerprinting of different hop samples used in the brewing trials (2018 vintage), acquired in the MIR region. As can be seen, the IR signature highlighted significant differences among samples, mainly in the spectral regions 3000–2800 cm^−1^, 1800–1400 cm^−1^ and <1100 cm^−1^. The first broad band located at about 3270 cm^−1^ was attributed to the O-H stretching, which was associated with the presence of water in the matrix and/or carboxyl groups present in compounds such as polyphenol acids, also reported in hop cones [13,23]. Two sharp peaks centered at 2916 and 2849 cm^−1^, related to the symmetric and asymmetric stretching of methylene groups, characteristics of lipids and fatty acids as well as of aromatics [24], were also visible in this region and they were more pronounced in the CAS_T samples (Figure 2b). Interestingly, the CAS_T samples were also characterized by a small but clear band (2954 cm^−1^) to the left of the intense signals due to the aliphatic stretching, which could be attributed to the stretching of =C-H of terminal group in non-conjugated alkenes as well as to the stretching of aromatic ring C-H bonds. The fingerprinting region between 1800 and 700 cm^−1^ showed significant differences between the two samples analyzed. The spectral band centered at 1735 cm^−1^ was attributed to the stretching of the C=O bonds in saturated esters and δ-lactones. Superimposing the graphs of the second-derivative IR spectra (Figure 4a,b; red lines) on the raw IR spectra in the range from about 1700 cm^−1^ to about 1400 cm^−1^, allowed the analysis of the overlapping peaks, such as amide I band components in the spectral region of 1690–1630 cm^−1^ and the bending absorption of NH bond (amide II) in the region 1570–1510 cm^−1^. As can be seen from Figure 4, these bands were present in the sample from Latium and absent in the sample coming from Tuscany, which showed far fewer absorption bands in this spectral range than the other sample analyzed (Figure 4b). From derivative spectrum of CAS_L sample (Figure 4a, red line), two bands at about 1580 cm^−1^ and around 1500 cm^−1^ were observed, highlighting the presence of conjugated aromatic rings. Besides, another two bands in the derivative spectrum of CAS_L sample (Figure 4b) centered at 1652 cm^−1^ and 1578 cm^−1^ were observed, which could be attributed to the absorption of the ring carbonyl bond and to the keto-enol carbonyl stretching vibrations of lupolones, respectively [25]. Both hop samples showed two sharp bands centered at about 1470 and 1460 cm^−1^ due to the stretching of the double aromatic bond. 

Table 1 shows the main chemical quality parameters of hop samples used in the brewing trials. Both total α- and β-acid content of the two hop samples analyzed were in line with the reported values for the Cascade variety (4.5–8.9% for α-acids; 3.6–7.5% for β-acids) [6,9]. The hop cones from Tuscany showed significant higher values of bitter acids compared to CAS_L ones (*p* < 0.05), highlighting a growing area effect on hop bitter acid content as also reported in the literature [6]. Interestingly, both hop samples showed a very low cohumulone content, in contrast to that reported by technical data sheets for Cascade variety and by international studies, in which it was always above 21% (on average 33–40%; [6,9]. Low levels of cohumulone are often associated with great beer foam stability and the so-called noble hops are characterized by a cohumulone ratio of 25% or less [26]. In the present study, both hop samples showed comparable and good values of HSI [8]. With regard to the total oil content (TOC), the CAS_T samples showed a significant lower level of TOC than the CAS_L ones, but still in line with the oil content of the Cascade variety grown in Italy [6]. According to Van Holle et al. [8], the aroma profile of hops is a valuable tool for the assessment of *terroir* influence. Herein, a detailed aroma profiling of hop hydrodistillates was obtained through the use of gas chromatographic analysis coupled with an olfactometric detector (GC-O analysis; Table 2), confirming the possible role of growing location on hop characteristics [8]. 

As regards hop hydrodistillates, 19 OEs were found in total in both samples analyzed (Table 2). In both cases the “resinous, beer” OE, associated with the hop prevalent compound β-myrcene, showed the highest area value and a middle (2) intensity. Another important OE (RT: 16–17 min) was described as “beer”, with a resinous and sulfurous note, but it was not clearly associated with any compound. Terpenes with an olfactory threshold under their limit of detection or sulfur compounds not detectable using an FID could have a role in determining this OE.

The monoterpene alcohols linalool and geraniol, together with phenyl ethanol, were responsible for the “floral”, “geranium” and “rose” OE. “Floral, geranium” showed higher intensity (2) and area in the CAS_T samples. The CAS_L profile showed three OEs not found in the CAS_T one, possessing “vegetable, burnt, spicy, resinous” notes, at the same retention time of major sesquiterpenes, α-humulene, β-caryophyllene and β-farnesene; it is not clear, however, if these compounds were responsible for those OEs, since they were present in both extracts in high quantity, but they were not always detectable due to their low olfactory thresholds [27].

**Figure 5:**
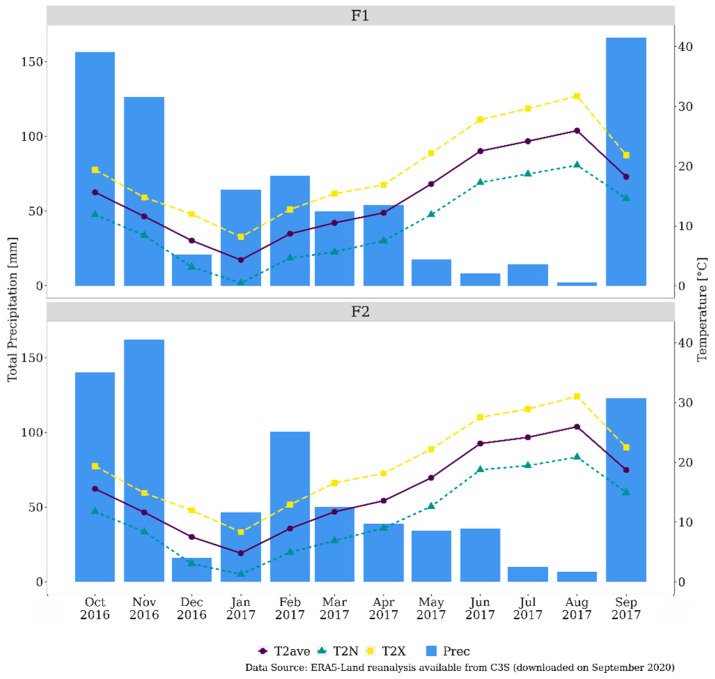
Weather conditions in the hop farms under investigation: monthly data of maximum (T2X), minimum (T2N), average (T2ave) temperature, and total precipitation (TP). Farm code: F1: Latium; F2: Tuscany.

The production and quality of hops strictly depend on weather conditions in the growing season. Even modest warming could affect both yields and quality [28]. In particular, extreme events, such as drought and heat waves, have been shown to have relevant impacts on hop production and α-acid contents [29]. As far as the present study is concerned, total precipitation was unevenly distributed and extremely concentrated during the autumn months, with a peak of monthly values over 160 mm for both sites. Besides, during the growing season (from March to August), the amount of precipitation was very limited (Figure 5). As regards temperature, the period showed extremely low values in January and high values for both sites in August, when the maximum temperature exceeded 31 °C. For the same period of analysis, Table 4 shows the distribution of the monthly temperature and precipitation anomalies (reference period: 1981–2010) estimated at the administrative regional level. It is worth noting that relevant positive anomalies up to 3 °C characterized the temperature from February to August, both in Latium and Tuscany, where the F1 and F2 sites are located, respectively. At the same time, precipitation showed extremely high negative anomalies (≤−90 mm) in December 2016 and negative values throughout most of the growing period (March–August), in the two regions, as already observed in Parisse et al. [30].

### 3.3. Brewing Trials: Evaluation of Hop Growing Area on Beer Quality Traits 

Hop characteristics are reflected in the flavor of derived beers [31]. Herein, the beers’ olfactometric profiles (Table 5), as expected, are characterized by OEs associated with the presence of fermentation products. Overall, acids and alcohols are responsible for “acid”, “fermented” and “chemical” notes, while esters are known to be responsible for “fruity” and “floral” notes [32]. As regards the present beers, it was not possible to identify the odorants responsible for all OEs; however, some differences were clear. The main OE in both profiles, recognized as characteristic by panelists, was described as “floral”, and it was associated with ethyl octanoate (Table 5 and Table 6). Similarly to the respective hop profile, the CAS_L beer profile presented one OE, which was not present in the CAS_T beer profile and they were related to the prevalent hop sesquiterpenes, indicating that the differences in hop olfactometric profiles due to the growing location are able to impart detectable differences in the derived products.

The GC analysis of beer samples allowed the identification of 45 compounds belonging to the following chemical groups: (1) compounds with furan structure; (2) phenolic compounds; (3) aldehyde compounds; (4) cyclic compounds of the “enonic” type, with five or six carbon atoms (e.g., maltol or cyclotene), (5) short or medium chain fatty acids.

It can be noted that for some compounds there were small differences (Table 7): CAS_T beer was characterized by the presence of a slightly higher quantity of linalool, geraniol (not identified in the CAS_L beer) and β-damascenone (responsible for odors as quince, peach) (*p* < 0.05). CAS_L beer, on the other hand, was characterized by the presence of a higher quantity of citronellol (rose, lemon), 2-phenylethylacetate (rose, honey), and 2-phenylethanol (*p* < 0.05). There were also larger amounts of cinnamic acid and ethyl cinnamate with spicy odors. Slight but not significant differences were also observed in the compounds myrcene, caryophyllene (major in the CAS_T beer) and humulene (major in the CAS_L beer).

Finally, the analytical differences found in the two beers were then confirmed by the sensory analysis, where the panel recognized a sensory difference between them. The tetrad test was statistically significant (*p* = 95%), since 9 of the 16 assessors grouped the samples in the correct way. The descriptors chosen were the volume and persistence of the foam, the intensity of amber yellow for the beer color, and intensity of turbidity. The attributes of the taste and of mouthfeel were sweetness, bitterness, acidity, body, carbonation, and taste—olfactory persistence.

**Figure 6:**
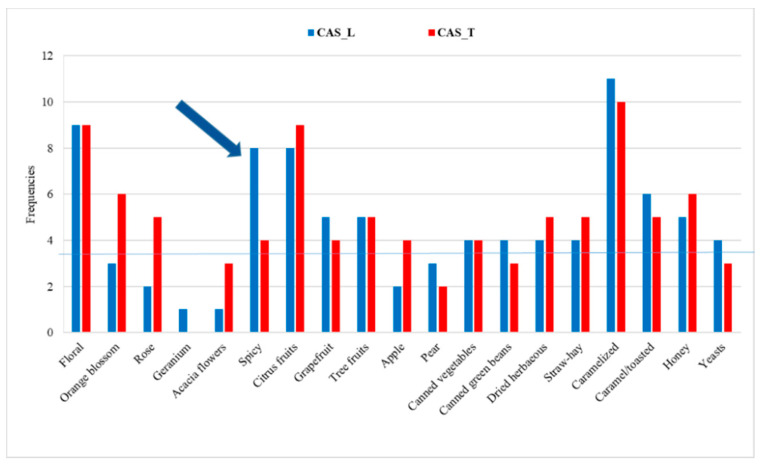
The frequencies of identification of odor attributes in the Latin and Tuscan beers.

Figure 6 shows the frequencies of the odor attributes identified by the panel, which were orange blossom, rose, grapefruit, pear, apple, canned green beans, straw-hay, honey, yeast, caramel/toasted. In the CAS_T beer, orange blossom and acacia flowers had a higher number of identifications, with spicy having a high number of identifications in the CAS_L beer, while being negligible in the CAS_T one. For this reason, it was not considered in the sensory profile. 

**Figure 7:**
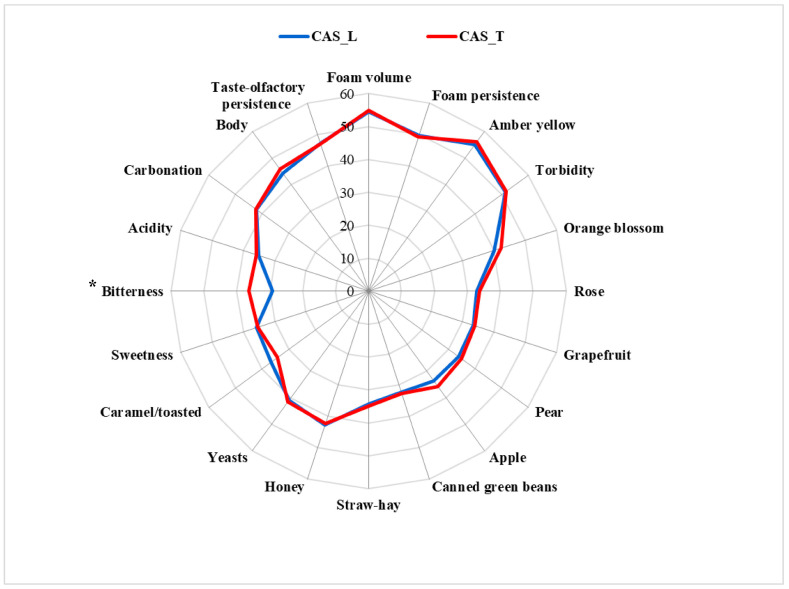
The sensory profiles of the Tuscan and Latin beers (average of three sessions). The asterisk points to the only attribute with a significative difference between the beers.

Figure 7 shows the sensory profile of the beers analyzed as the average of the three repetitions. Only the descriptor “bitter” was statistically significant (*p* = 95%) and its levels were higher in the CAS_T beer than in the CAS_L one. These results agree with the cohumulone content of the hop samples used, which was statistically higher in the CAS_T hops than in the CAS_L ones (19.45 and 18.28%, respectively; Table 1).

## 4. Conclusions

Herein, we analyzed the possibility of transferring the concept of *terroir* to the craft beer chain, by analyzing the link between the same hop cultivar from different geographical origins and the derived beers in terms of chemical and sensorial features. Beers brewed with hops cv Cascade grown in Latium and Tuscany showed significant statistical differences, both from an analytical and sensorial point of view, in bitterness intensity and in their spicy aroma.

These preliminary results need further investigation, including soil analysis of different growing areas, information that was not possible to obtain from this study. Nevertheless, they point out that hops of the same cultivar grown in different regions express distinctive aroma and molecular fingerprinting profiles, and that this is most likely attributable to an effect of *terroir*, thus becoming a driving force for craft brewers seeking to replace imported hops by exploiting the rural distinctiveness of their community in their beers.

## Figures and Tables

**Table 1:** Main features of hop farms and hop samples.

Farm Code	Geographical Coordinates	Region	Hop Variety	Sample Code	α-Acids (% *w*/*w*)	β-Acids (% *w*/*w*)	Cohumulone Ratio (% rel) ^1^	HIS ^2^	Total Oil Content (mL 100 g^−1^)	TPC (mg GAE g^−1^) ^3^
F1	41°63′46″ N-12°87′18″ E	Latium	Cascade	CAS_L	5.23 ± 0.01 a	6.34 ± 0.02 a	18.28 ± 0.01 a	0.30 a	1.50 ± 0.05 b	60.6 ± 0.1 a
F2	43°35′18″ N-10.31′19″ E	Tuscany	Cascade	CAS_T	7.19 ± 0.01 b	7.33 ± 0.01 b	19.45 ± 0.01 a	0.28 a	0.90 ± 0.04 a	66.6 ± 0.6 b

^1^ Relative cohumulone is expressed as cohumulone to total α-acids percent ratio; ^2^ HSI: hop storage index; ^3^ TPC: total polyphenol content, data are expressed as mg gallic acid equivalents per g of hop sample (on dry basis). In a column, different letters indicate significant differences (*p* < 0.05).

**Table 2:** GC-O analysis of hop samples: odour descriptors, average peak areas, and maximum odour intensities.

	Hop Origin		Tuscany		Latium	
OE ^1^	KI ^2^	Descriptor	Area	*I_max_*	Area	*I_max_*
1	778	Alcohol, pungent	616	1	698	1
2	780	Herbaceous, floral	1653 a	1	0 b	-
3	840	Green, plastic	2706 a	2	0 b	-
4	883	Floral	2872	1	547	1
5	905	Beer	13,657	2	9825	2
6	921	Earth, vegetable	3281	2	0	-
7	928	Sulfur, herbaceous	7250	2	2969	1
8	963	Acid, rancid	5893	2	4802	2
9	980	Floral, resinous	12,628 a	3	0 b	-
10	992	Resinous, beer	27,462	3	14,549	2
11	1122	Pungent, terpene	5241	2	6259	1
12	1126	Floral, rose	10,422	2	9500	2
13	1211	Floral, terpene	1338	1	1089	1
14	1228	Floral	1633	1	1813	1
15	1248	Floral, geranium	4024	2	1596	1
16	1278	Sweet, fat, floral	5988	2	6540	1
17	1377	Floral	0	-	1892	1
18	1402	Vegetable, resinous	0	-	3559	1
19	1490	Spicy, resinous	0	-	2957	1

^1^ OE: odorous event; ^2^ Kováts Index calculates using a linear series of *n*-alkanes. Differences between mean area values followed by different letters on the same row are significant (*p* < 0.05, LSD test); -: not detectable.

**Table 3:** Compounds identified by GC-MS in CAS_T and CAS_L hop oils.

KI ^1^	MW ^2^	Compound	Main Fragments ^3^	A_u_^4^ CAS_L		A_u_^4^ CAS_T	
				AV	ST DEV	AV	STD DEV
780	100	Hexanal	56(100), 44(100), 41(90), 57(90),42(50), 72(35)	2.0	0.1	3.5	0.1
853	98	(*E*)-2-Hexenal	41(100), 55(90), 69(90), 83(80), 98(30)	1.5	0.1	3.8	0.9
939	136	α-Pinene	93(100), 77(30), 12(20), 105(20), 136(20)	0	0	2.4	3.3
980	136	β-Pinene	93(100), 69(25), 41(25), 79(25), 121(20), 136(15)	38.6	9.4	112.9	6.3
990	136	β-Myrcene	93(100), 69(50), 41(50), 79(15), 53(15), 136(10)	1287.8	67.2	2127.9	71.1
1032	158	Amyl isobutyrate	43 (100), 70 (80) 71 (70) 55 (30) 89 (20)	4.2	0.5	10.9	0.6
1036	136	Limonene	93(100), 68(50), 77(40), 136(30), 121(20)	24.7	3.0	54.6	1.6
1040	136	β-Ocymene	93(100), 91(50), 79(40), 78(35), 77(30), 136(10)	6.1	1.0	6.1	0.2
1085	154	Linalool	71(100), 93(85), 41(50), 55(45), 80(40), 121(35)	53.3	7.3	91.8	1.1
1217	152	Methyl salicylate	120(100), 92(60), 152(50), 121(30), 65(15)	10.9	1.7	13.7	0.2
1224	172	Methyl nonanoate	74(100), 87(50), 129(20), 141(20), 172 tr	6.3	0.1	6.5	0.0
1250	154	Geraniol	69(100), 41(55), 93(10), 123(10), 154 tr	7.7	0.6	22.4	0.2
1277	152	(*Z*)-Citral (Neral)	69(100), 41(70), 84(30), 94(25), 137(25), 152(10)	10.4	2.2	11.9	0.2
1281	170	2-Undecanone	58(100), 43(100), 71(90), 59(80), 85(10), 170 tr	9.2	1.1	16.2	0.1
1291	184	Methyl-(*Z*)-4-decenoate	74(100), 110(75), 55(50), 67(55), 152(50)	28.8	1.2	37.4	0.1
1300	182	(*E*)-Methyl geraniate	69(100), 41(40), 114(40), 123(30), 83(20), 182 tr	12.8	1.1	43.3	0.5
1360	196	Neryl acetate (*Z*-)	69(100), 93(50), 41(50), 42(45), 80(20)	6.7	0.3	8.5	0.1
1380	196	Geranyl acetate (*E*-)	69(100), 43(45), 93(40), 121(25), 136(20), 196 tr	69.9	0.9	70.5	0.9
1411	204	*trans*-Caryophyllene	93(100), 133(100), 79(70), 69(65), 204(10)	652.8	28.2	677.8	5.3
1437	204	Germacrene D	161(100), 105(45), 91(40), 119(30), 133(10), 204(10)	27.0	1.8	31.2	0.3
1438	204	α-Bergamotene	119(100), 93(95), 41(30), 107(35), 79(30), 204 tr	30.1	1.7	37.3	0.1
1445	204	α-Humulene	93(100), 80(30), 121(30), 147(25), 107(20), 204(10)	1341.4	35.4	1510.4	11.6
1450	204	α-Amorphene	161(100), 119(50), 105(55), 91(45), 79(40), 204(30	73.2	4.0	83.9	1.1
1490	204	β-Selinene	105(100), 93(95), 79(80), 121(60), 161(60), 204 (65)	96.3	4.8	114.9	0.7
1500	204	α-Farnesene	93(100),41(50), 69(50), 107(50), 79(45), 107(45), 204 (10)	21.4	0.8	25.1	0.4

^1^ Kováts Index, calculated using a *n*-alkanes linear series; ^2^ Molecular Weight; ^3^ relative quantitation, assuming as 100 the most abundant fragment; ^4^ area units (area/10^6^).

**Table 4:** Weather conditions in the hop farms under investigations. (a) Monthly data of temperature and precipitation; (b) Monthly anomalies of the same variables (reference period 1981–2010), referred to the correspondent administrative regions.

**(a)**															
**Farm Code**	**Variable**		**2016**			**2017**									**Whole Period**
			**October**	**November**	**December**	**January**	**February**	**March**	**April**	**May**	**June**	**July**	**August**	**September**	
F1	Temperature (°C)	T2N	11.9	8.4	3.1	0.5	4.6	5.6	7.5	11.9	17.3	18.7	20.2	14.6	10.4
		T2X	19.4	14.8	12	8.2	12.8	15.4	16.9	22.2	27.8	29.7	31.7	21.9	19.4
		T2ave	15.6	11.6	7.5	4.3	8.7	10.5	12.2	17	22.5	24.2	25.9	18.2	14.9
	Precipitation (mm)	TP	156.4	126.3	21.1	64.1	73.5	49.9	53.9	17.5	8.2	14.2	2.2	166.2	754
F2	Temperature (°C)	T2N	11.7	8.4	3.1	1.3	4.9	6.9	9	12.6	18.8	19.4	20.9	14.9	11.0
		T2X	19.4	14.9	12	8.3	12.9	16.6	18.1	22.2	27.5	28.9	31	22.5	19.5
		T2ave	15.6	11.6	7.5	4.8	8.9	11.7	13.6	17.4	23.1	24.2	26	18.7	15.3
	Precipitation (mm)	TP	140.1	162.1	16.1	46.7	100.6	50.1	39.1	34.2	35.4	9.9	6.8	123	764
**(b)**															
**Region**	**Variable**		**2016**			**2017**									
			**October**	**November**	**December**	**January**	**February**	**March**	**April**	**May**	**June**	**July**	**August**	**September**	
Latium	Temperature (°C)	T2N	0.2	1.1	−0.4	−2.0	2.9	1.8	0.4	0.6	2.5	1.1	2.1	−0.6	
		T2X	−0.1	0.8	1.6	−1.5	2.6	2.7	1.3	1.4	3.1	1.5	3.4	−1.4	
	Precipitation (mm)	TP	46.5	−14.5	−103.7	−9.6	−12.7	−33.8	−39.6	−38.2	−31.5	−6.0	−33.6	85.3	
Tuscany	Temperature (°C)	T2N	0.1	1.3	0.0	−1.5	3.1	2.2	0.8	0.7	2.3	0.8	1.8	−0.3	
		T2X	−0.2	0.8	1.6	−1.1	2.2	3.1	1.9	1.1	2.9	1.1	3.1	−1.2	
	Precipitation (mm)	TP	29.6	41.5	−90.4	−28.1	31.0	−32.2	−40.3	−27.0	−17.8	−15.9	−39.5	43.1	

T2X: maximum temperature, T2N: minimum temperature, T2ave: average temperature, TP: total precipitation.

**Table 5:** GC-O analysis of beer samples: odour descriptors, average OE areas and maximum odour intensities (median, *I_max_*).

	Hop Origin		Toscana		Lazio	
OE ^1^	KI ^2^	Descriptor	Area	*I_max_*	Area	*I_max_*
1	596	Chemical, fruity	647	1	487	1
2	875	Floral, vegetable	364 a	2	0 b	-
3	903	Chemical, fuel	1053 a	3	0 b	-
4	980	Sweet, fruit	706	2	501	1
5	1012	Acid, fermented	652 a	2	0 b	-
6	1066	Fermented, vegetable	494	2	1186	1
7	1184	Chemical, floral	0 b	-	590 a	1
8	1190	Floral	1010	1	1040	2
9	1278	Floral, spicy	636	1	804	1
10	1342	Floral, sweet	0 b	-	228	1
11	1402	Herbaceous, hay	528	1	554	1
12	1470	Floral	0 b	-	187 a	2

^1^ OE: odorous event; ^2^ RT: retention time (min). Differences between mean area values followed by different letters on the same row are significant (*p* < 0.05, LSD test). “-“: not detectable.

**Table 6:** Compounds identified by GC-MS and quantified by FID in CAS_T and CAS_L beers SPME headspace.

KI ^1^	MW ^2^	Compound	Main Fragments ^3^	CAS_L (mg L^−1^)		CAS_T (mg L^−1^)	
600	88	Ethyl acetate	45(100), 61(100), 70(80), 88(50)	1.05	0.35	0.89	0.15
720	88	1-butanol 3-methyl	55(100), 70(80), 57(25), 56(10), 88 tr	5.16	1.33	3.79	0.70
896	130	Ethyl pentanoate	88(100), 85(95), 57(70), 60(40), 101(30)	0.13	0.01	0.07	0.03
980	136	Ethyl hexanoate	88(100), 99(50), 60(40), 101(30), 73(25)	2.89	0.71	4.49	0.84
992	144	Beta-myrcene	93(100), 69(80), 79(20), 107(5), 121(5), 136 tr	1.76	0.78	2.92	0.30
1085	154	Linalool	71(100), 93(90), 55(60), 80(30), 121(20)	0.08	0.04	0.12	0.04
1090	122	Phenethyl ethanol	91(100), 92(70), 122(30), 65(10), 77 tr	3.19	0.76	2.60	0.87
1180	172	Ethyl octanoate	88(100), 101(40), 127(35), 57(20), 70(20)	24.03	10.39	36.82	7.29
1212	146	β-citronellol	69(100), 55(50), 82(40), 95(35), 123(25)	0.28	0.05	0.12	0.03
1239	164	β-phenethyl acetate	104(100), 91(20), 78(5), 65(5), 51 (5)	0.16	0.11	0.37	0.06
1360	198	Ethyl-9-decenoate	55(100), 88(90), 69(70), 110(50), 101(45)	0.92	0.39	2.19	0.42
1380	200	Ethyl decanoate	88(100), 101(50), 73(30), 55(25), 156(20)	7.13	2.49	8.29	1.33
1425	204	*trans*-Caryophyllene	93(100), 133(100), 79(70), 69(65), 204(10)	0.08	0.02	0.2	0.01
1455	204	α-Humulene	93(100), 80(30), 121(30), 147(25), 107(20), 204(10)	0.30	0.07	0.74	0.06
1590	228	Ethyl dodecanoate	88(100), 101(50), 55(20), 73(20), 157(10)	11.24	3.22	1.53	0.27

^1^ Kováts Index, calculated using a *n*-alkanes linear series; ^2^ molecular weight; ^3^ relative quantitation, assuming as 100 the most abundant fragment.

**Table 7:** Average contents of aromatic compounds (μg L^−1^) present in CAS_L beer and CAS_T beer.

Compound (µg L^−1^)	Class of Compound	RT ^1^	LRI (Literature)	CAS_L Beer (Average)	Mean Absolute Deviation	CAS_T Beer (Average)	Mean Absolute Deviation	Pr > F(Model)
Myrcene	I	12.76	1174	21.3	2.4	37	11.1	n.s.
Isoamyl alcohol	A	13.66	1206	4876.0	331.2	4007.5	788.8	n.s.
Ethylhexanoate	EE	14.99	1232	63.4	54	116.2	21.5	n.s.
Ethylactate	EE	18.78	1334	11.1	1.2	18.1	6.3	n.s.
Hexanol	A	19.07	1360	99.1 a	4.7	184.1 b	15.4	0.034
3-ethoxypropanol	A	20.00	1375	15.1	1.5	25.9	5.7	n.s.
cis-3-hexenol	A	20.32	1390	30.9	1.1	65.8	13.9	n.s.
Ethyloctanoate	EE	22.4	1436	523.3	93.8	543.9	73	n.s.
1-octen-3-ol	A	22.86	1458	17.5	2.2	11	4.4	n.s.
2-acetylfuran	K	25.41	1500	1.1	0.2	0.9	0	n.s.
Benzaldehyde	AL	26.24	1529	1.9	0.2	1.7	0.3	n.s.
1.3-butanediol	A	26.45	1558	49.3	6	65.1	16.7	n.s.
Linalool	I	26.74	1555	127.5 a	2.2	143.9 b	1.2	0.024
Octanol	A	28.97	1564	26.6	14.9	9.1	1.2	n.s.
β-caryophyllene	I	29.03	1607	13.2	11.6	25.1	0.5	n.s.
Isobutyric acid	AC	30.05	1568	16.5	1.8	21.6	4	n.s.
Gamma-butyrolactone	L	30.45	1643	398.2 b	77.7	24.5 a	24.5	0.044
Ethyldecanoate	EE	30.75	1645	390.7	79.8	236	19.7	n.s.
Acetophenone	K	31.10	1660	13.4	5	2.5	2.5	n.s.
Phenylacetaldehyde	AL	31.14	1663	nd	Nd	2.4	2.1	n.s.
Furfurilalcohol	A	31.23	1678	10.3	1.8	14.7	2.6	n.s.
Isovaleric acid	AC	31.59	1672	175.5	11.6	184.6	5.2	n.s.
Alpha umulene	I	31.82	1665	87.5	16.2	61.5	10.5	n.s.
Methionol	A	33.39	1727	5.1	0	17.2	6	n.s.
Citronellol	I	35.00	1804	140.5	0.2	52.9	37.5	n.s.
2-phenylethyl acetate	E	36.00	1815	145.2	11.9	105.3	21.5	n.s.
Ethyldodecanoate	EE	36.65	1835	0	0	25.4	15.4	n.s.
β-damascenone	I	37.70	1832	203.8 a	0	495 b	33	0.013
Hexanoic acid	A	38.03	1855	2 b	0.2	1.1 a	0	0.037
3-ethylthiopropanol	A	38.26	1802	13.9 b	2.9	0 a	0	0.040
Geraniol	I	38.47	1861	0 a	0	19.6 b	4.3	0.045
Benzyl alcohol	A	39.22	1874	17.4	1.4	15.2	1	n.s.
2-phenylethanol	A	40.96	1922	15.1 b	0	5.8 a	0.2	0.000
2-methyl-2-pentenoic acid	AC	41.75	1909	493.7	173.8	290.5	14.3	n.s.
Octanoic acid	AC	45.58	2092	6411.5	768.9	2877.2	348.8	n.s.
Ethylcinnamate	EE	47.65	2145	31.6	0.5	18.4	6.6	n.s.
Nonanoic acid	AC	48.51	2168	109.7	6.9	50.4	17.4	n.s.
Eugenol	PH	48.67	2169	2	2	7.7	0.2	n.s.
4-vinyl-2-methoxyphenol	PH	49.63	2180	914.8 b	15.2	393.2 a	48.7	0.009
Decanoic acid	AC	51.88	2269	2350.5 b	64.6	808.3 a	35.8	0.002
8-acetoxylinalol	I	52.76	2362	299.4 b	10.8	110.2 a	15.2	0.010
9-decenoic acid	AC	53.52	2369	493.9	75	204.2	33.6	n.s.
4-vinylphenol	PH	55.29	2379	132.3 b	13.2	63.6 a	1.7	0.035
Lauric acid	AC	57.68	2503	441.6 b	6.2	186.1 a	7.6	0.001
3-hydroxybeta damascone	I	59.12	2559	12,827.5 b	260.2	5879.5 a	486.5	0.006
Phenylacetic acid	AC	60.07	2582	57.4 b	2.4	20 a	1.5	0.006
Methylvanillate	E	61.14	2600	14.3	4.6	4.5	1	n.s.
Acetovanillone	L	62.24	2664	41.1 b	5.4	15.2 a	0.9	0.042
Myristic acid	AC	63.77	2692	77.6	16.9	30.5	2.3	n.s.
Propiovanillone	L	64.30	2719	50.1 b	4.8	12.8 a	0.7	0.016
Vanillin	B	68.63	2601	4.6	1.6	2.0	0.7	n.s.
Cinnamic acid	AC	70.80	2835	147.4	16.9	70.4	17.9	n.s.
Ethylvanillate	EE	70.86	2658	65.3	4.9	34.1	13.1	n.s.
Palmitic acid	AC	72.85	2886	513	112.6	120.8	20.6	n.s.
Oleic acid	AC	74.76	3172	99.3	89.6	88.4	12.7	n.s.
Homovanillic acid	AC	77.16	3099	62.6	12	18.1	2.2	n.s.

^1^ RT: retention time (min). Different letters are statistically different (*p* < 0.05) between samples in the same line. A = alcohols; AC = acids; AL = aldehydes; E = esters; EE = ethyl esters; B = benzenoids; PH = phenols; I = isoprenoids; K = ketones. n.s.: not significant.

## Data Availability

All data is contained within the article.
